# Sex-Specific Morphological and Neuromuscular Profiles of U-15 Colombian Basketball Players

**DOI:** 10.3390/jfmk10040422

**Published:** 2025-10-29

**Authors:** Alex Ojeda-Aravena, María Alejandra Camacho-Villa, Fernando Millan-Domingo, Ronald Quintero-Bernal, Jeimy Andrea Merchán, Fabio Villafrades, Adrián De la Rosa

**Affiliations:** 1 Departamento de Ciencias de la Actividad Física, Universidad de Los Lagos, Osorno 5290000, Chile; alex.ojeda@ulagos.cl; 2 Programa de Investigación en Deporte, Sociedad y Buen Vivir (DSBv), Universidad de Los Lagos, Osorno 5290000, Chile; 3 Harpeer Research Group, Yumbo 760001, Colombia; alejacvilla@gmail.com (M.A.C.-V.);; 4 Pain Study Group (GED), Physical Therapy School, Universidad Industrial de Santander, Bucaramanga 680002, Colombia; 5 Freshage Research Group, Department of Physiology, Faculty of Medicine, University of Valencia, Centro de Investigación Biomédica en Red Fragilidad y Envejecimiento Saludable (CIBERFES), Fundación Investigación Hospital Clínico Universitario/INCLIVA, 46010 Valencia, Spain; fernando.millan-domingo@uv.es; 6 Physical Activity and Sport Program, Unidades Tecnológicas de Santander, Bucaramanga 680006, Colombia; 7 Body, Physical Activity and Sport Study Group (GECAFD), Sports Department, Universidad Industrial de Santander, Bucaramanga 680002, Colombia; villafra@uis.edu.co

**Keywords:** team sports, adolescents, somatotype, body composition, athletic performance

## Abstract

**Background:** Basketball performance is highly dependent on morphological and neuromuscular traits, especially during adolescence, when rapid growth and maturation generate marked sex-based differences. However, limited data are available on Latin American players’ performance. This study aimed to compare the anthropometric characteristics, body composition, somatotype, and neuromuscular performance of male and female Colombian U-15 national basketball players. **Methods:** The sample consisted of thirty-seven players (20 males, 17 females; mean age: 14.8 ± 0.4 years) during the preparatory phase of the 2022 South American U-15 Championships. Anthropometry and body composition were evaluated following ISAK standards, and somatotype was calculated using Carter and Heath’s method. Neuromuscular performance included countermovement and squat jumps, bilateral handgrip strength, and isometric knee extensor and flexor peak torques. Between-sex differences were examined using *t*-tests, Welch’s tests, or Mann–Whitney U tests. The effects of sex on body composition, somatotype, and neuromuscular outcomes were assessed using MANOVA. **Results**: Males had higher muscle mass, lower adipose mass, and greater limb lengths than females (*p* < 0.01). No sex differences were observed in BMI, waist or hip circumference, or quadriceps strength. Regarding neuromuscular performance, males exhibited higher handgrip strength, hamstring torque (absolute and relative), and jump performance than females. **Conclusions:** Males showed greater muscle mass, strength, and jump performance, whereas females displayed higher fat levels and endomorphy than males. These findings provide useful data for optimizing training load prescriptions, guiding targeted strength programs, and developing sex-specific strategies for injury prevention and talent identification in adolescents.

## 1. Introduction

Basketball is an intermittent team sport characterized by a combination of high-intensity actions, such as accelerations, decelerations, changes in direction, jumps, lateral sliding, and static efforts, alternating with lower-intensity activities [1]. Studies have shown that junior male basketball players spend 22.57% of their total game time in high-moderate intensity activities and 63.28% in low-intensity zones [2,3]. Male and female elite basketball players change movement types every 1–3 s and perform an average of 44 ± 7 jumps per game [4]. Moreover, specific basketball movements (e.g., rebounding, sprinting, dribbling, shooting, and blocking) are performed at high intensities and are strongly associated with the development of strength, power, speed, and agility [5].

Adolescence is a critical period for athletic development and is marked by rapid growth and maturation processes that differ by sex. These biological differences affect physical performance, body composition, and neuromuscular capabilities, highlighting the importance of considering sex-specific characteristics in the assessment and training of young basketball players [6].

Anthropometric profiles and body composition play decisive roles in basketball performance. An adequate profile, characterized by greater height and lower fat mass, can provide significant advantages for the court [6]. Previous studies have demonstrated that a lower body fat percentage (BF%) is positively associated with competitive level and negatively related to the performance of explosive actions, such as sprinting, changes in direction, and vertical jumps [7,8,9]. Furthermore, dimensions such as body height, arm length, and upraised arm height are correlated with enhanced performance in basketball-specific tasks, such as obstacle dribbling and speed-related actions [10]. Furthermore, a correlation between morphology, physical capabilities, and game performance, through performance indices derived from match statistics, has been documented in elite female basketball [11].

A complementary approach to body composition assessment is the use of somatotypes, which provide a more comprehensive description of an athlete’s physique by categorizing body structure into endomorphic, mesomorphic, and ectomorphic components [10]. These outcomes allow for a deeper understanding of how morphology influences basketball performance, offering valuable insights for designing tailored training programs and identifying morphological differences between male and female players [12].

In addition to morphology, basketball performance relies heavily on strength and power. Lower limb strength and explosive capacity are essential for game action, including jumping, rebounding, and blocking. The countermovement jump (CMJ) and squat jump (SJ) are widely used to assess explosive strength in basketball, providing insight into the contribution of the stretch-shortening cycle and pure concentric power, respectively [13,14]. Upper-body strength is also critical, with handgrip strength (HGS) serving as a practical indicator of muscular capacity that directly influences ball control, passing, and defensive actions. Upper and lower limb strength contribute decisively to overall basketball performance, with greater jumping and grip capacities consistently observed in players with higher competition levels [11].

A rigorous assessment of morphological and neuromuscular variables during adolescence is essential to underpin athletic development, guide training prescription, and optimize long-term performance trajectories. These variables are also significantly associated with basketball performance and talent progression. Anthropometric characteristics (e.g., stature, somatotype) and neuromuscular outputs (e.g., jump height, torque) discriminate selection levels and positional demands, and they explain variance in key performance indicators and game outcomes. These associations have been documented in elite youth cohorts and in meta-analytic syntheses on talent identification, reinforcing the utility of an integrated anthropometric–motor profile along developmental pathways [15].

Regarding the neuromuscular profile, and acknowledging its value, most evidence relies on vertical and horizontal jump tests to evaluate power performance. Measuring isometric knee torque adds joint specificity, in-field reliability, and the capacity to characterize strength components (peak and rate of torque development, RTD) that are not captured by jump tests or by the handgrip rate of torque development [16]. Integrating these measures with the squat jump (SJ) and countermovement jump (CMJ) yields a more robust neuromuscular profile that is useful for selection, individualized prescription, and decision-making in injury prevention and return to sport [17,18].

Despite its importance, sport and sex-specific normative references for youth basketball remain scarce in Latin America. Although age and sex-stratified reference values exist in other regions (e.g., federated Tunisian basketball) and in mixed cohorts from sports academies, Latin American datasets are fragmentary. This gap hinders benchmarking, talent identification, and load progression in the region, and justifies the development of local percentile charts and reference tables (by sex, age band, and playing position). This knowledge gap limits the ability to optimize training methodologies and to compare developmental trajectories with those observed in other populations.

Therefore, the present study aimed to compare anthropometric characteristics, body composition, and neuromuscular performance, including jump performance, HGS, and isometric lower limb strength, between young Colombian male and female basketball players.

## 2. Materials and Methods

### 2.1. Subjects

This cross-sectional analytical study was conducted during the preparatory phase for the 2022 South American U-15 Women’s and Men’s Championships. A total of 37 youth basketball players from Colombian U-15 national teams participated, comprising 17 females (mean age: 14.9 ± 0.24 years) and 20 males (mean age: 14.8 ± 0.44 years). All athletes who participated in the training microcycle were eligible for inclusion in the study.

Participants were healthy and free from any medical conditions that could affect performance, hand function, anthropometry, or daily activities. All procedures were explained in detail to the athletes and their legal guardians, and non-participation had no effect on team selection. Written informed consent was obtained from the parents or legal guardians, and assent was provided by all athletes. The study was conducted in accordance with the Declaration of Helsinki and approved by the University Ethics Committee (Approval No. 0010-2022; 2 May 2022).

### 2.2. Testing Procedures

Comprehensive details regarding the anthropometric parameters and neuromuscular performance are provided in the subsequent section. All assessments were performed before the training session in the laboratory of the University during morning hours (between 8:00 and 11:00 a.m.), on two separate days for females and males.

All the evaluations took place in a temperature-controlled environment and followed a predefined sequence: anthropometric measurements, Countermovement Jump (CMJ), Squat Jump (SJ), Handgrip Strength (HGS); and maximal isometric strength of the knee extensors and flexors.

Lower and upper limb dominance were determined by asking athletes which arm or leg they would use to throw/kick a ball [19,20].

Assessments were carried out by three researchers, each with nine years of experience in sports research. Anthropometric data were gathered by a level 2 anthropometrist, achieving an intra-rater intraclass correlation coefficient (ICC) between 0.91 and 0.96, indicating excellent reliability.

HGS, CMJ, and SJ were evaluated by a second researcher, trained in participant positioning and standardized verbal encouragement. Intra-rater ICC values for HGS were 0.98 for the dominant hand and 0.97 for the non-dominant hand. For CMJ and SJ, ICC values ranged from 0.94 to 0.95, indicating high reliability [21]. The maximal isometric strength test of the knee extensors and flexors was performed by a third researcher, who received specific training on participant positioning, device use and software protocols. The ICC values for these measures were 0.85 and 0.87, respectively.

### 2.3. Anthropometric Measurements and Body Composition

Anthropometric measurements were performed in accordance with the standards of the International Society for the Advancement of Kinanthropometry (ISAK). Participants wore minimal clothing and were barefoot to optimize measurement accuracy.

Stature was measured to the nearest 0.1 cm using a stadiometer (Seca^®^ 274, Hamburg, Germany; Technical Error of Measurement = 0.019%), and body mass was measured to the nearest 0.1 kg using a TANITA BC 240 MA (Tanita Corporation, Arlington Heights, IL, USA). Eight skinfold thicknesses were assessed with a skinfold caliper (Cescorf, Porto Alegre, Brazil) at the following ISAK defined sites: triceps, bíceps, subscapular, supraspinale, iliac crest, abdominal, front thigh, and medial calf [22].

Five girths were measured with a metal tape (Cescorf, Porto Alegre, Brazil; measurement range of up to 100 cm and accuracy to 0.1 cm) at the following sites: relaxed arm, flexed arm, waist, hip, and calf. Bone breadths on both sides (humerus, bistyloid, and femur) were measured to the nearest 0.1 cm using a small bone anthropometer (Cescorf, Porto Alegre, Brazil). Upper limb lengths on both sides (arm and forearm length, hand breadth, hand length, and first-to-fifth finger distance) were recorded to the nearest 0.1 cm with a segmometer (Cescorf, Porto Alegre, Brazil), following previously described protocols [7,15].

Based on the measurements, body composition was estimated using the calculations proposed by De Rose and Guimaraes following their four-compartment model (fat mass, bone mass, muscle mass, and residual mass). BF% was calculated using the Yuhasz [23] equation adapted for adolescent athletes; fat mass was calculated as the product of BF% and body mass, divided by 100; bone mass from the bicondylar breadths of the humerus and femur [24]; residual mass as a fixed proportion of body mass [25]; and muscle mass by subtraction from total body mass. Likewise, the somatotype of these athletes was determined based on the model proposed by Heath and Carter, obtaining the value of the three components: endomorphic, mesomorphic, and ectomorphic. The analysis was performed using a Microsoft Excel spreadsheet.

The sum of six skinfold measurements (triceps, subscapular, supraspinale, abdominal, front thigh, and medial calf) and the sum of eight skinfold measurements (triceps, bíceps, subscapular, supraspinal, iliac crest, abdominal, front thigh, and medial calf) were also calculated. The estimated upper arm muscle area was calculated for the dominant side using the equation proposed by Frisancho [26].

All measurements were taken on the right side of the body by the same ISAK Level 2 anthropometrist to minimize inter-observer variability.

### 2.4. Neuromuscular Performance Assessment

*Vertical Jump Tests*

The CMJ and SJ were used to evaluate lower-limb neuromuscular performance using a contact platform (Chronojump Boscosystem, Barcelona, Spain) following standardized protocols described by Bosco et al. [27].

Before testing, athletes completed a five-minute warm-up consisting of light jogging, skipping, and dynamic exercises (half-squats, lunges, and leg swings). They were familiarized with the jumping technique with three submaximal practice trials of each type (CMJ and SJ) before performing maximal attempts.

For the CMJ, participants began in an upright standing position with hands placed on the hips to eliminate arm swing, performed a rapid downward movement to approximately 90° of knee flexion, and immediately executed a maximal vertical jump. For the SJ, participants started from a static squat position at ~90° of knee flexion, held the position for 2–3 s to minimize the contribution of the stretch-shortening cycle [28], and then jumped vertically without countermovement. Verbal encouragement was provided during testing to ensure maximal effort.

Three valid trials were performed for each jump type, separated by two minutes of passive recovery. Players were asked to jump as high as possible, and the highest value for each type of jump was used for analysis.

*Handgrip Strength Assessment*

Maximal HGS was measured bilaterally using a digital handheld dynamometer (Takei 5401; Tokyo, Japan) with a measurement accuracy of 0.1 kg. Participants were tested in a standing position, with the shoulder of the test arm adducted and the elbow flexed at 90°. The forearm and wrist were maintained in a neutral position to ensure proper alignment between the hand and forearm during grip assessment. The dynamometer was individually adjusted to each participant’s hand size to ensure proper flexion of the metacarpophalangeal joints. Before testing, standardized verbal instructions were provided, and verbal encouragement was given throughout the procedure to elicit maximal effort [8].

Three maximal voluntary contractions were performed per hand, each lasting 3–5 s. A 60 s rest interval was implemented between trials to minimize potential fatigue. HGS values were recorded in kilograms (kg), and the highest value from the three trials, for each hand, was used for statistical analysis [22].

*Maximal Isometric Lower Limb Strength*

Maximal isometric strength of the quadriceps and hamstring muscles was assessed at 60° of knee flexion, with 0° representing full knee extension. Measurements were conducted using a portable hand-held dynamometer (Chronojump Boscosystem, Barcelona, Spain). This method has been demonstrated to be reliable for assessing lower-limb strength in both general and athletic populations [29].

**Figure 1:**
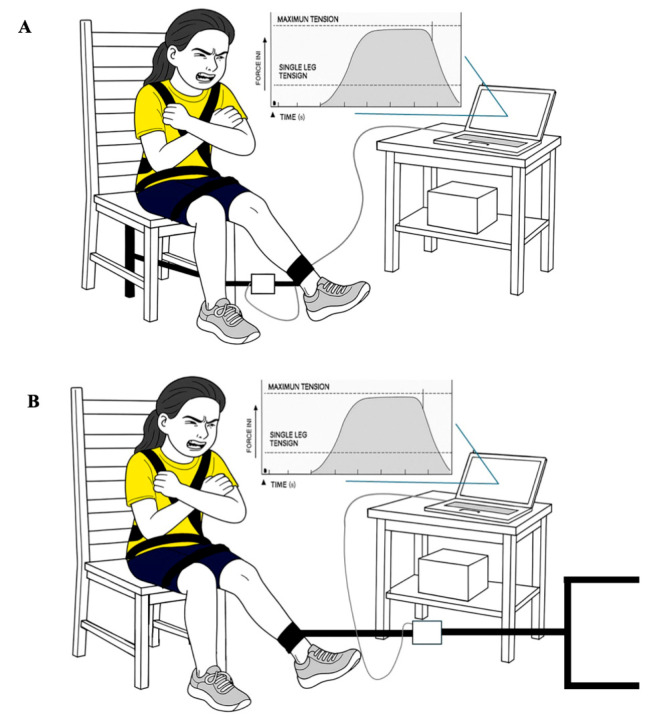
Set up of the maximal isometric lower limb strength. (**A**) Quadriceps and (**B**) hamstrings.

Before testing, players completed a standardized, sport-specific warm-up as detailed elsewhere [30]. In addition, they were familiarized with the protocol by performing two submaximal contractions for both flexion and extension in both legs. After the warm-up, testing occurred with participants seated on an adjustable chair, with hips and knees stabilized by an adjustable and rigid strap around the thighs and hips to minimize trunk compensations (Figure 1). A knee flexion angle of 60° was verified using an electronic goniometer (K-force Sens, Kinvent, France). Figure 1 of the protocol was refined using the AI image generator Plus tool (Image Generator GPT (ChatGPT, version GPT-4o)) to enhance visual clarity.

For the quadriceps assessment, the dynamometer was attached to a rigid bar anchored to the back of the chair and connected to the tested leg’s ankle via a strap positioned approximately 1–2 cm above the malleolus. The strap length was adjusted to achieve 60° of knee extension (Figure 1A). In contrast, during hamstring assessment, the dynamometer was attached to a rigid bar anchored to the wall in front of the chair, again using a malleolus level strap adjusted to ensure 60° of knee flexion (Figure 1B).

To avoid compensatory movements from the non-tested leg, participants were instructed to keep it relaxed (i.e., do not anchor the leg to the chair). Given that the original protocol does not allow support on the chair or any object, participants were asked to place their hands on the opposite shoulder (Figure 1). The dynamometer data were transmitted to an A/D converter and collected at 160 Hz, then filtered and smoothed using Chronojump software (v2.5.2-64, Chronojump Bosco System, Barcelona, Spain) according to manufacturer recommendations.

Participants performed two maximal isometric contractions per movement for both lower limbs, each lasting ~5 s, with 60 s rest intervals between trials. They were verbally encouraged to exert maximal effort throughout the test.

Torque was calculated by multiplying the measured force by the moment arm, which was defined as the linear distance from each participant’s lateral epicondyle of the femur to the center of the ankle strap connected to the load cell. The highest peak torque value recorded for each muscle group was used for the analysis. Additionally, the relative peak torque was calculated by normalizing the absolute value to each participant’s weight. Both values were included in the statistical analysis.

### 2.5. Statistical Analysis

All statistical analyses were performed using Jamovi software (v2.6, https://www.jamovi.org). Descriptive statistics were calculated for all variables and presented as mean ± standard deviation (SD) or median and interquartile range (IQR), as appropriate. The Shapiro–Wilk test was used to assess univariate normality, and Levene’s test was applied to verify the homogeneity of variances.

Between-sex differences were examined using independent sample *t*-tests for normally distributed variables with homogeneous variances, and Mann–Whitney *U* tests for non-normally distributed variables. Effect sizes were reported as Cohen’s d for *t* test and rank-biserial correlation (r) for the Mann–Whitney U test. For interpretation, we used conventional benchmarks: *d* ≈ 0.20 (small), 0.50 (medium), 0.80 (large); and *r* ≈ 0.10 (small), 0.30 (medium), 0.50 (large) [31].

Two separate multivariate analyses of variance (MANOVA) were performed to assess the effect of sex on (1) body composition variables and (2) neuromuscular performance variables. The assumptions for MANOVA were tested using Box’s M test for homogeneity of covariance matrices and Shapiro–Wilk’s test for multivariate normality. When multivariate normality was violated, Pillai’s Trace was used as the multivariate test statistic due to its robustness to assumption violations.

Significant multivariate effects were followed by univariate analysis for each dependent variable. Partial eta squared (η^2^p) was calculated as an estimate of the effect size for each univariate test and interpreted according to Richardson guidelines: small (0.01 to 0.05), medium (0.06 to 0.13), and large (≥0.14) [32]. The significance level was set at *p* < 0.05, with Bonferroni adjusted thresholds applied where appropriate.

## 3. Results

Table 1 summarizes the results of the analysis of anthropometric variables by sex. Multiple comparisons with the *t*-test and Mann-Whitney U test, with a Bonferroni correction, revealed significant sex differences in most variables. Males showed notably greater height (*p* < 0.001, d = 1.58), waist-to-hip ratio (*p* < 0.001, d = 1.64), and a wide range of bone breadths, including the humerus, femur, bistyloid, and hand widths (*p* < 0.001; *d* = 1.53–3.47) on both sides of the body (Table 1). Significant differences were also observed in limb lengths, with males exhibiting longer arms, forearms, hands, and finger lengths bilaterally (*p* < 0.001; d = 1.37–2.20 or r > 0.73).

On the other hand, female players exhibited significantly higher values in all skinfold thicknesses, including triceps, subscapular, supraspinale, abdominal, front thigh, and medial calf (*p* < 0.001; d = 1.21–2.54), as well as in the sum of 6 and 8 skinfolds (*p* < 0.001, *d* = 2.26 and 1.89, respectively), indicating greater subcutaneous fat (Table 2).

Several variables did not show statistically significant differences after Bonferroni correction (Table 2), including body weight (*p* = 0.014), BMI (*p* = 0.210), waist circumference (*p* = 0.066), hip circumference (*p* = 0.141), relaxed arm girth (*p* = 0.028), calf girth (*p* = 0.079), and the ileocrestal skinfold (*p* = 0.090).

Overall, these findings highlight similar and substantial sex-based differences in body composition and structural dimensions among adolescent basketball players. Males generally displayed greater skeletal breadths and linear measurements, whereas females showed higher values in subcutaneous fat measures.

After studying the anthropometric profile, a MANOVA was conducted to assess the effect of sex on body composition, the somatotype components and the somatotype attitudinal mean (SAM). The overall multivariate effect was significant, Pillai’s Trace = 0.97, *F*(10,26) = 76.40, *p* < 0.001, indicating marked sex-based differences in body composition profiles between male and female players (Table 2). The univariate analyses revealed that females presented significantly higher values for body fat percentage and adipose mass, whereas male showed greater muscle mass, residual mass, bone mass, and upper arm muscle area (Table 2).

**Figure 2:**
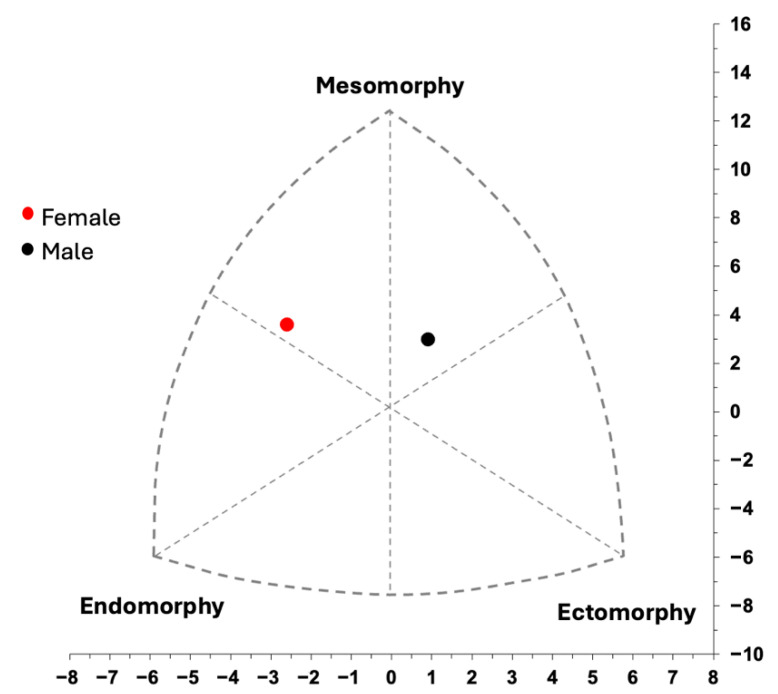
Somatotype distribution (somatochart) of young male and female basketball players.

Regarding somatotype components, females demonstrated markedly higher endomorphy values (*F* = 51.49, *p* < 0.001), while males scored significantly higher in ectomorphy. Mesomorphy tended to be higher in females, although this difference was not statistically significant (*F* = 3.57, *p* = 0.067). Thus, while male participants exhibited a meso-ectomorphic somatotype (2.36–3.85–3.05), with ranges of 1.40–3.94 for endomorphy, 1.50–6.90 for mesomorphy, and 0.86–5.93 for ectomorphy. Female participants demonstrated a meso-endomorphic somatotype (4.18–4.64–1.88), ranging from 2.78 to 5.75 for endomorphy, 2.58–6.39 for mesomorphy, and 0.56–4.42 for ectomorphy. To facilitate interpretation, individual somatotypical values are illustrated in Figure 2.

Table 3 presents the results of the analysis of muscular strength and power output variables by sex. A MANOVA revealed a statistically significant overall multivariate effect (Pillai’s Trace = 0.90, F(12,21) = 16,3, *p* < 0.001), indicating a strong influence of sex on physical performance variables.

Follow-up univariate analyses showed that males achieved higher HGS values than females in both (*F* = 22.69, *p* < 0.001, *η*^2^*p* = 0.41) and non-dominant hand (*F* = 46.98, *p* < 0.001, *η*^2^*p* = 0.59) hands, with very large effect sizes, reflecting superior upper body strength in male players.

In quadriceps performance, although males recorded higher peak torque values in both sides, the differences were not statistically significant (*p* = 0.46 and *p* = 0.34, respectively). In contrast, hamstring peak torque was significantly greater in males than females on both the dominant (*F* = 12.04, *p* < 0.01, *η*^2^*p* = 0.27) and non-dominant sides (*F* = 9.43, *p* < 0.01, *η*^2^*p* = 0.23.

The largest differences emerged in jump performance, where males outperformed females in both the CMJ and SJ height. These differences were statistically significant (*F* = 55.62 and 41.57, respectively; *p* < 0.001 for both) and accompanied by large effect sizes (*η*^2^*p* = 0.63 and 0.56), highlighting a clear disparity in explosive lower-limb power between sexes.

## 4. Discussion

### 4.1. Sex-Based Differences in Anthropometric and Body Composition Profiles

This study investigated sex-based differences in anthropometric characteristics, body composition, and neuromuscular performance among U-15 Colombian national basketball players. Here, we provide reference values of anthropometric and neuromuscular performance variables in highly trained players. Similarly, our findings showed significant sex-based differences in most variables assessed, highlighting the relevance of considering sex-specific profiles when evaluating youth athletes and designing development programs.

Basketball is highly dependent on physical and structural traits, particularly during adolescence [33,34], a critical period for growth and motor development. While several studies have documented anthropometric variations according to competitive level, to our knowledge, this is the first study to explore sex-specific differences in both anthropometric and neuromuscular characteristics among Colombian adolescent basketball players [35].

### 4.2. Anthropometric Profile and Body Composition

Significant sexual dimorphism arises during puberty, not only in the timing of pubertal milestones but also in changes in body composition [35,36]. Although both sexes experience a rapid increase in body fat during this period, the pattern and extent of fat accumulation differ between males and females. Changes during puberty include increased linear growth and muscle development in males, and greater fat accumulation and earlier skeletal maturity in females [37]. In boys, rising testosterone levels drive significant increase in bone length, height, and muscle mass, along with a decrease in limb fat [38]. In contrast, girls show a smaller increase in height and muscle mass, but a significant increase in body fat deposition [39].

Previous studies have reported significant differences in stature, arm span, leg length, and hand length among young basketball players across different levels of expertise [40]. Similarly, sex-specific differences in body fat percentage and body composition have also been documented in young athletes across different sports, including basketball [41].

Consistent with the above-mentioned reports, male players in our study exhibited significantly greater height, bone breadths, and limb lengths, especially in the arms, forearms, and hands, compared to females (Table 2). These morphological traits, especially longer arms, forearms, and hands, align with prior findings in elite young male basketball players [42,43,44]. Such advantages are critical in basketball, where reach, hand size, and stature directly influence shooting, passing, rebounding, defensive efficiency, and physical performance in a broad range of tests [45,46,47].

In terms of body composition, several studies have shown that male athletes typically display a more mesomorphic somatotype, characterized by greater muscularity and a more robust physique. In contrast, female athletes tend to present a higher endomorphic component, reflecting a greater proportion of body fat and a softer overall body composition [48,49]. In our study, female players had significantly higher values in skinfold thicknesses, endomorphy, body fat percentage, and adipose mass, indicating a greater accumulation of both subcutaneous and total fat. Conversely, male players exhibited more ectomorphic profiles and total muscle mass, denoting a leaner and more linear body shape (Table 3). This pattern aligns with typical sex-specific physiological development during adolescence, as documented in the studies of young athletes [50,51]. In basketball-specific contexts, it has been reported that female youth and professional players tend to display high endomorphy and low ectomorphy, which may affect performance and efficiency in specific actions within the sport [52,53]. On the other hand, the absence of significant differences in SAM values, which reflects the overall deviation from a balanced somatotype, suggests similar levels of somatotype dispersion in both sexes.

### 4.3. Neuromuscular Performance

It is well known that men’s athletic performance exceeds that of women, especially in power sports, due to their greater strength, speed, and endurance [54,55]. This physical advantage arises during early adolescence, when male puberty begins. Afterwards, men acquire bigger muscle mass, greater strength, larger and stronger bones, and higher circulating hemoglobin. They also experience mental and/or psychological differences [56].

Muscle strength is a key component of athletic performance. In many sports, maximal repeated jumps are necessary, and it has been shown that there is a direct relationship between knee flexor and extensor muscle strength and jumping performance, subsequently leading to sports success [57,58]. In team sports, including basketball, lower limb strength is a critical determinant of performance, as it supports key actions such as sprinting, jumping, accelerations and decelerations, and physical duels during both offensive and defensive situations [59,60,61,62]. Athletes across all playing positions must perform shuffling movements at varying intensities during several match actions [63]. This demands the ability to perceive and react to opponents’ movements rapidly, placing significant demands on their agility, lateral displacement, and acceleration capacities [64].

This study identified notable sex-based differences in almost all neuromuscular performance variables studied. Males exhibited higher hamstring isometric strength than females, whether measured as absolute or relative peak torque on the dominant or non-dominant side (Table 3). Interestingly, quadriceps strength values did not differ significantly between the sexes. This pattern aligns with previous studies indicating that sex-related differences in lower limb strength become more pronounced in the hamstring, widening with maturation, while quadriceps strength remains relatively comparable between the sexes during adolescence in isometric conditions [65].

Owoeye et al. [6] reported that although males generally achieve higher absolute strength in both muscle groups, the disparity between sexes is greater for the hamstrings, potentially due to sport-specific demands that preferentially stimulate hamstring development in males, hormonal influences, and differences in muscle fiber composition.

In the present study, the differences observed in the posterior chains may be linked to a greater reliance on hamstring-driven actions in boys during matches and training. Many studies have documented that male elite junior basketball players cover more high-intensity distance, perform more sprints and jumps, and engage in more deceleration activities than their female counterparts [66]. Because these actions typically require the primary participation of this muscle group, it is plausible to think that males have achieved greater adaptation. Moreover, differences could be explained, in part, by the contribution of maturation in our athletes. As documented, before, during, and after puberty, boys are on average stronger than girls [67]. The progression in physical performance during adolescence is typically slower and smaller in magnitude in girls compared to boys. This has been reported in both team and individual sport athletes [68], as well as in the general population [69]. This difference is largely attributed to a greater increase in fat mass in girls and muscle mass in boys during puberty and adolescence [70], as found in the present study.

Along with this, during adolescence, boys produce higher levels of circulating testosterone (about 15–20-fold more than females [71]), which has been associated with the increased growth velocity [72] and reduced adipose accumulation in males compared to females [73]. Higher testosterone levels, in turn, result in more muscle mass, which facilitates greater strength production and more advantageous ground reaction forces during high-intensity actions. In contrast, at this stage, girls produce higher levels of circulating estrogen, which are about 4-fold-higher among females than males [72]. This hormone has limited anabolic effects, and it is not a primary contributor to the large sex differences in athletic performance [66].

Regarding jumping performance, we found that male players significantly outperformed female players in both the CMJ and SJ jumps’ height (*p* < 0.001), with moderate to large effect sizes (d = 0.63 and 0.57, respectively). These findings reflect sex differences in explosive power and stretch-shortening cycle utilization, which are critical for high-intensity basketball actions like jumping, rebounding, and quick take-offs [74]. Results in this study are consistent with those reported in adolescent and professional players of different team sports, including basketball. These differences may be attributed to morphological characteristics of the muscle between males and females, such as muscle thickness, pennation angle, and fascicle length, which tend to favor males in producing greater muscular strength [52]. Moreover, it has been described that the muscle area occupied by fast-twitch fibers is greater in males than in females [75], which is associated with greater strength [76].

On the other hand, in the present study, we also found significant sex-based differences in HGS for both the dominant and non-dominant side, with male players demonstrating higher values than their female counterparts (*p* < 0.001). The effect sizes were small to moderate (d = 0.41 and 0.59, respectively). However, no significant differences were found between the dominant and non-dominant sides in boys or girls.

While not a basketball-specific action, HGS has been associated with several movements that rely on the continuous use of wrist and digit flexors. These technical actions include catching, control, passing accuracy, shooting performance, speed dribbling, and time in an obstacle dribbling performance, in young and adult basketball players [77,78]. Results in our study align with prior research reporting superior upper-limb strength in adolescent male athletes, largely explained by greater muscle mass development and neuromuscular efficiency during puberty [79].

Our results were also higher than those reported in other male and female young and adult athletes across different sports, including basketball [80], but lower than those reported in Chilean professional players, and Italian and Greek adolescent basketball players [52,77,81]. The morphological differences in upper limbs have previously been described by our research group as a key factor in the HGS performance. Thus, athletes with larger hand breadth, hand length, upper arm length, and arm circumference display higher HGS [8]. Aligning with these previous reports, the results in the present study demonstrated that these anthropometric measures were larger in boys than in girls (Table 1). In addition, the estimated upper arm muscle area in our study was higher in boys than in girls (Table 2), which has also been related to greater HGS on both the dominant and non-dominant sides [22]. Thus, differences found in HGS could also be explained, in part, by the anthropometric characteristics of the sample.

In addition, as described above, greater testosterone production in males could modulate the force production in favor of boys. Recently, a study conducted in 641 Chinese male adolescents investigated the associations of sex steroids with muscle parameters [82]. The results indicated that the concentrations of serum testosterone and free testosterone were positively related to HGS. Researchers also found that this relationship was only observed in the late-post pubertal group (15.9 years), suggesting a potential threshold effect. Because the age of male players in our study is 14.9 years, these findings reinforce the idea that sex differences reported here could be strongly explained by the activating effects of sex hormones.

The present investigation offers a detailed characterization of the anthropometric, body composition, and neuromuscular performance profiles of a homogeneous sample of U-15 Colombian national basketball players. Our findings provide valuable insight into sex-specific physical and performance traits that may influence basketball development at this competitive stage. Nevertheless, caution should be exercised when extrapolating these results to athletes at different competitive levels, age categories, or training backgrounds, as the sample consisted exclusively of highly trained players engaged in national team preparation.

The sample size was determined by the availability of players selected for the preparatory phase of the South American U-15 Championships. Moreover, the cross-sectional nature of the study precludes any inferences about causality or longitudinal changes in these variables. While chronological age was controlled for, biological age or maturity status was not assessed, which could have provided an additional context for interpreting sex differences during adolescence. Additionally, the exclusive use of isometric strength assessment for knee muscles limits the ability to extrapolate to dynamic, sport-specific contexts.

Future research should employ longitudinal designs to monitor the evolution of anthropometric, body composition, and neuromuscular performance profiles during adolescence, as well as their relationship with performance metrics and injury risk in basketball. Incorporating dynamic strength tests, movement efficiency assessments, and match play performance analyses would provide a more holistic understanding of the sex-specific demands and adaptations in youth basketball.

## 5. Conclusions

This study provides novel, sex-specific data on anthropometric characteristics, body composition, and neuromuscular performance in U-15 Colombian national basketball players. Males presented greater height, skeletal breadths, and limb length, as well as higher HGS, hamstring strength, and vertical jump performance. The females showed higher subcutaneous fat levels and adiposity indicators. No significant sex-specific differences were found in quadriceps isometric strength; this may reflect similar developmental patterns for this muscle group in highly trained athletes during adolescence. These findings align with known growth and maturation processes, highlighting the influence of both biological factors and sport-specific demands on performance profiles. The reference values generated can inform coaches, strength and conditioning staff, and sports medicine professionals in designing targeted, sex-specific training and injury prevention strategies for highly trained young basketball players.

## Figures and Tables

**Table 1:** Anthropometric profile of young male and female basketball players.

Variable	Male	Female	Statistic (t/U)	Effect Size (d/r)
Basic				
Height (m)	1.79 (0.08)	1.66 (0.07) ***	t = 5.02	d = 1.58
Weight (kg)	71.64 (9.85)	64.12 (7.49)	t = 2.58	d = 0.85
BMI (kg/m^2^)	22.35 (2.34)	23.31 (2.22)	t = −1.28	d = 0.43
Waist-to-hip ratio	0.79 (0.03)	0.74 (0.03) ***	t = 4.98	d = 1.64
Skinfolds (mm)				
Triceps	8.80 (3.33)	14.88 (3.12) ***	t = −5.70	d = 1.91
Subscapular	8.70 (1.67)	12.21 (3.73) ***	t = −3.58	d = 1.21
Biceps	5.08 (1.49)	7.15 (1.52) ***	t = −4.18	d = 1.38
Ileocrestal	11.20 (4.05)	14.74 (6.28)	U = 114.0	r = 0.32
Supraspinal	7.58 (1.82)	13.18 (3.88) ***	t = −5.47	d = 1.86
Abdominal	11.45 (3.52)	18.03 (4.32) ***	t = −5.11	d = 1.67
Front thigh	10.88 (3.13)	20.21 (4.77) ***	t = −7.13	d = 2.35
Medial calf	7.55 (2.01)	15.32 (3.71) ***	t = −7.73	d = 2.54
Sum of 6 skinfolds	54.95 (13.87)	93.82 (19.86) ***	t = −6.78	d = 2.26
Sum of 8 skinfolds	71.23 (18.51)	115.71 (26.68) ***	t = −5.79	d = 1.89
Girths (cm)				
Waist	74.34 (3.54)	71.69 (4.91)	t = 1.90	d = 0.63
Hip	94.07 (5.16)	96.64 (5.19)	t = −1.51	d = 0.51
Arm (relaxed)	27.99 (2.42)	26.29 (1.98)	t = 2.30	d = 0.75
Arm (flexed and tensed)	30.63 (1.96)	27.33 (2.20) ***	t = 4.82	d = 1.59
Calf	37.95 (5.01)	35.64 (1.72)	t = 1.93	d = 0.64
Lengths (cm)				
Arm length	34.23 (1.94)	31.72 (1.50) ***	t = 4.34	d = 1.44
Forearm length	27.73 (2.61)	24.35 (1.52) ***	U = 22.5	r = 0.86
Hand length	20.30 (1.22)	18.85 (4.33) ***	U = 40.5	r = 0.76
First-to-fifth finger distance	22.75 (1.43)	20.73 (1.27) ***	U = 45.5	r = 0.73
Arm length (left)	34.05 (2.07)	31.44 (1.75) ***	t = 4.10	d = 1.37
Forearm length (left)	27.08 (2.08)	24.08 (1.37) ***	t = 5.24	d = 1.75
Hand length (left)	20.38 (1.23)	17.93 (1.00) ***	t = 6.53	d = 2.17
First-to-fifth finger dist. (left)	23.13 (1.64)	20.91 (1.07) ***	U = 32.0	r = 0.81
Bone breadth (cm)				
Humerus	7.25 (0.38)	6.40 (0.33) ***	t = 7.15	d = 2.38
Femur	8.96 (1.19)	10.01 (0.58) ***	U = 54.5	r = 0.67
Bistyloid	6.67 (1.00)	5.58 (0.31) ***	t = 4.60	d = 1.53
Hand	9.72 (0.65)	7.98 (0.35) ***	t = 10.41	d = 3.47
Humerus (left)	7.32 (0.48)	6.48 (0.31) ***	t = 6.16	d = 2.05
Bistyloid (left)	6.19 (0.29)	5.58 (0.25) ***	t = 6.84	d = 2.27
Hand (left)	8.79 (0.43)	7.98 (0.35) ***	t = 6.64	d = 2.20

All data are presented as mean (standard deviation). *** denotes *p* < 0.001 vs. male; t/U: differences were assessed with *t*-test/Mann-Whithey U test; d/r: effect size was assessed with Cohen’s d and rank-biserial correlation.

**Table 2:** Somatotype characteristics of young male and female basketball players.

Variable	Male	Female	F	Effect Size (η^2^p)
Body Fat % (Yuhasz)	8.36 (1.46)	18.10 (3.07) ***	159.27	0.82
Adipose mass (kg)	6.09 (1.83)	11.76 (3.12) ***	47.19	0.57
Muscle mass (kg)	35.51 (4.49)	27.21 (2.79) ***	38.53	0.52
Residual mass (kg)	17.26 (2.37)	14.11 (1.75) ***	20.51	0.37
Bone mass (kg)	12.78 (1.83)	11.04 (1.33) **	11.37	0.24
Upper arm muscle area (cm^2^)	50.82 (6.65)	37.38 (5.49) ***	43.85	0.56
Endomorphy	2.36 (0.64)	4.18 (0.90) ***	51.49	0.60
Mesomorphy	3.85 (1.31)	4.64 (1.21)	3.57	0.09
Ectomorphy	3.05 (1.24)	1.88 (1.09) **	9.13	0.21
SAM	1.64 (1.91)	1.31 (0.54)	1.65	0.04

All data are presented as mean (standard deviation). **/*** denotes *p* < 0.01/*p* < 0.01 vs. male.

**Table 3:** Sex-Based Differences in Neuromuscular Performance Variables.

Variable	Male	Female	F	η^2^p
Handgrip Strength (kgf)				
Dominant	39.61 (7.13)	29.06 (5.24) ***	22.69	0.41
Non dominant	40.41 (6.63)	26.66 (4.77) ***	46.98	0.59
Quadriceps Performance				
Peak Torque (N·m)_DS_	177.01 (65.24)	145.17 (38.68)	2.48	0.07
Relative Peak Torque (N·m·kg^−1^)_DS_	2.51 (0.90)	2.27 (0.58)	0.56	0.02
Peak Torque (N·m)_NDS_	186.66 (63.68)	155.09 (39.87)	3.27	0.09
Relative Peak Torque (N·m·kg^−1^)_NDS_	2.64 (0.89)	2.40 (0.61)	0.93	0.03
Hamstring Performance				
Peak Torque (N·m)_DS_	155.11 (20.98)	121.14 (9.69) ***	33.89	0.51
Relative Peak Torque (N·m·kg^−1^)_DS_	2.21 (0.27)	1.91 (0.23) ***	12.04	0.27
Peak Torque (N·m)_NDS_	161.53 (10.98)	121.14 (9.69) ***	19.58	0.09
Relative Peak Torque (N·m·kg^−1^)_NDS_	2.30 (0.42)	1.90 (0.27) ***	9.43	0.23
Jump Performance (cm)				
CMJ height	34.80 (5.64)	23.28 (3.05) ***	55.62	0.63
SJ height	32.02 (5.95)	21.31 (3.04) ***	41.57	0.56

All data are presented as mean (standard deviation). *** denotes *p* < 0.01 vs. male, respectively.

## Data Availability

The datasets used and/or analyzed during the current study are available from the corresponding author upon reasonable request.
